# Effect of high pressure treatment on the aging characteristics of Chinese liquor as evaluated by electronic nose and chemical analysis

**DOI:** 10.1038/srep30273

**Published:** 2016-08-03

**Authors:** S. M. Zhu, M. L. Xu, H. S. Ramaswamy, M. Y. Yang, Y. Yu

**Affiliations:** 1 College of Biosystems Engineering and Food Science, Zhejiang University; Key Laboratory of Equipment and Informatization in Environment Controlled Agriculture, Ministry of Agriculture, 866 Yuhangtang Road, Hangzhou 310058, China; 2 Department of Food Science, McGill University, 21111 Lakeshore Road, St-Anne-de-Bellevue, QC H9X 3V9, Canada

## Abstract

Several high pressure (HP) treatments (100–400 MPa; 15 and 30 min) were applied to Chinese “*Junchang*” liquor, and aging characteristics of the liquor were evaluated. Results from the principal component analysis and the discriminant factor analysis of E-Nose demonstrated that HP treatment at 300 and 400** **MPa resulted in significant (p < 0.05) changes in aroma components of the liquor. An increase in total ester content and a decrease in total acid content were observed for all treated samples (p < 0.05), which was verified by gas chromatography analysis. In addition, a slight decrease in alcohol content was found for HP treatment at 400 MPa for 30 min. These changes and trends were in accordance with the natural aging process of Chinese liquor. However, HP treatment caused a slight increase in solid content, which might be somewhat undesirable. Sensory evaluation results confirmed that favorable changes in color and flavor of Chinese liquor were induced by HP treatment; however, overall gaps still existed between the quality of treated and six-year aged samples. HP treatment demonstrated a potential to accelerate the natural aging process for Chinese liquor, but long term studies may be needed further to realize the full potential.

As listed in Wikipedia, the earliest evidence of alcohol in China is traced to jars from Jiahu which date to about 7000 BC. This early rice mead was produced as a fermented product from rice, honey, and fruit^1^. Chinese liquor is an alcoholic beverage produced as a distillate of traditional ferment from grains. Owing to its mellow taste and full aroma, the liquor has been one of the most popular alcoholic beverages for centuries with a consumption of more than 4 million kiloliters each year worldwide, creating an yearly sales revenue of over 500 billion Chinese Yuan^2^. The top brands of Chinese liquors include: Maotai, Wuliangye, Xifeng, Shuanggou Daqu, Yanghe Daqu, Gujing Tribute, Jinnanchun, Luzhou Laojiao Tequ, Fen, and Dong. Typical manufacturing process for the liquor generally involves the primary fermentation, distillation, aging and, if required, blending^3^. The principal grain used for manufacturing Chinese liquor is sorghum or a mixture of sorghum, wheat, rice and corn. These raw material(s) is (are) milled, cooked and then mixed with Daqu powder (starter culture). After several months of fermentation, the liquor is distilled out using steam distillation process. Upon distillation, the liquor (young, raw or fresh) often has some undesirable harsh flavor, and so the freshly distilled liquor is usually aged for several years to refine the flavor and develop the bouquet aroma^4^. Therefore, aging plays an indispensable role in the process of producing high-quality Chinese liquor.

Since the liquor aging process is usually very long, alternate technologies are often sought to shorten the required aging time. In recent years, several methods based on physical or chemical modification have been reported in literature for liquor aging. Zhang *et al*.^5^ investigated the effect of pulsed electric field treatment on the composition of phenolic compounds of brandy during aging in oak barrels. Their results demonstrated that phenolic compounds such as tannins, total phenols as well as volatile phenols significantly increased after the pulsed electric field treatment, as commonly observed during aging. Thus it was recommended that electric field treatment could possibly used to accelerate the aging process. Chang *et al*.^6^ used 20 kHz ultrasonic waves to accelerate the aging of different wines, and then related the changes in pH, alcohol content, gas chromatography measurements and sensory evaluation scores to the aging process. They observed that the ultrasonic treatment helped to age the rice wine more rapidly, with similar quality, than standard aging; however, they also noticed that the treatment did not accelerate the aging of maize wine with comparable quality. Schwarz *et al*.^7^ employed oak chips and ultrasound, as extraction method, to accelerate the aging of Brandy de Jerez on a laboratory scale. After 30 days, samples obtained by the developed method were reported to possess similar analytical and sensorial characteristics to those which had been aged in the traditional way for an average time of between 6 and 18 months.

During the past decade, the application of high pressure (HP) treatment for food processing and preservation has increased rapidly. HP processing is currently used worldwide for various food processing applications to give products of higher quality that were hitherto not possible^8^,^9^,^10^,^11^. HP processing has also been used in wine processing. Tao *et al*.^12^ studied the effect of HP treatment on the physicochemical and sensorial properties of red wine. They reported that HP significantly influenced the chromatic characteristics and the phenolic composition of wine after treatment at 650 MPa for 0.25, 0.5, 1 and 2 h. Moreover, HP treatment for 2 h at 650 MPa was reported to significantly reduce the intensities of sour and fruity odor of wine. In another research, Tabilo-Munizaga *et al*.^13^ reported that HP treatment resulted in modification of α-helical and β-sheet structures of wine proteins. HP treatment has been proved to be beneficial for accelerating the ripening of cheese^14^. However, to our best knowledge, the effect of HP on the aging of liquors, especially Chinese liquor, has not been studied yet.

The objective of this study was therefore to evaluate the effect of HP treatment on the major volatile flavor components of a kind of Chinese liquor (*Junchang* brand, which is a popular local brand, produced by Junchang Liquor Factory in Sichuan province) subjected to 15–30 min treatment at 100–400 MPa. Volatile profile analysis with an electronic nose (E-Nose), four chemical tests, namely total acid content, alcohol content, total ester content and total solid content, and gas chromatography, were conducted to compare the profiles from treated and untreated liquors and the liquor stored for 6 years. In addition, sensory evaluation was also performed.

## Results

### PCA results

As defined generally in Wikipedia, the Principal Component Analysis (PCA) is “a statistical procedure that uses an orthogonal transformation to convert a set of observations of possibly correlated variables into a set of values of linearly uncorrelated variables called principal components. The number of principal components is less than or equal to the number of original variables. This transformation is defined in such a way that the first principal component has the largest possible variance (that is, accounts for as much of the variability in the data as possible), and each succeeding component in turn has the highest variance possible under the constraint that it is orthogonal to the preceding components”. PCA is a type of multivariate statistical method, which data-converses and dimensionality-reduces the extracted multi-index information of sensors. It linearly classifies the characteristic vector after dimensionality reduction and displays the major two-dimensional map on a PCA analysis map. In the case of the absence or lack of sample information, PCA can quickly scan all data to determine the associated features of the samples and make a conclusion based on the available information^15^,^16^,^17^.

**Figure 1:**
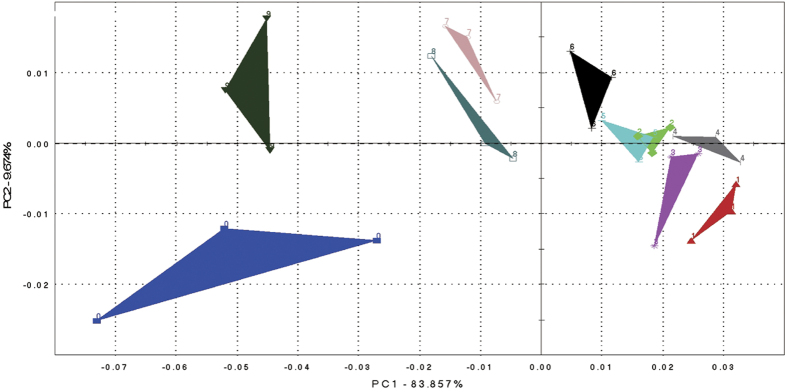
PCA map of 9 samples: six year aged (0), control (1), 100 MPa-15 min (2), 100 MPa-30 min (3), 200 MPa-15 min (4), 200 MPa-30 min (5), 300 MPa-15 min (6), 300 MPa-30 min (7), 400 MPa-15 min (8), 400 MPa-30 min (9).

The classification results of different groups through PCA analysis are shown in Fig. 1. PC1 and PC2 were taken as coordinate axes for the PCA analysis on samples, and it was found that the linear combination of PC1 and PC2 explained an overall variance higher than 93%. The figure also shows that except for samples 2 and 5, the different groups of liquor components were well-separated from each other in the selected coordinate system. Furthermore, response values of samples 7, 8, 9 and 0 which referred to 300 MPa-30 min, 400 MPa-15 min, 400 MPa-30 min treatment and six year aged liquor respectively, were negative on PC1, while the other groups were positive. Since most of the 18 sensors used in the electronic nose were placed on PC1 (explained by an overall variance of 83.9%), this result implied that samples 7, 8, 9 and 0 could be clustered as one category, while samples 1 to 6 are clustered as another category. Meanwhile, an apparent difference between sample 0 (six-year aged liquor) and samples 7, 8, 9 in PC2 can also be observed in Fig. 1 indicating that some difference still remained between these variables.

### DFA results

Discriminant Function Analysis (DFA) is another multivariate technique for describing a mathematical function that will distinguish among predefined groups of samples. DFA has a strong connection to multiple regression and principal components analysis. In addition, DFA is the counterpart to ANOVA: in DFA, continuous variables (measurements) are used to predict a categorical variable (group membership), whereas ANOVA uses a categorical variable to explain variation (prediction) in one or more continuous variables. DFA is a classification technique that optimizes the distinguishing ability of variables by recombining sensor data^18^. With mathematical manipulation, it minimizes the differences between data of the same type and broadens the disparities between data from different categories to establish a data recognition model^19^.

**Figure 2:**
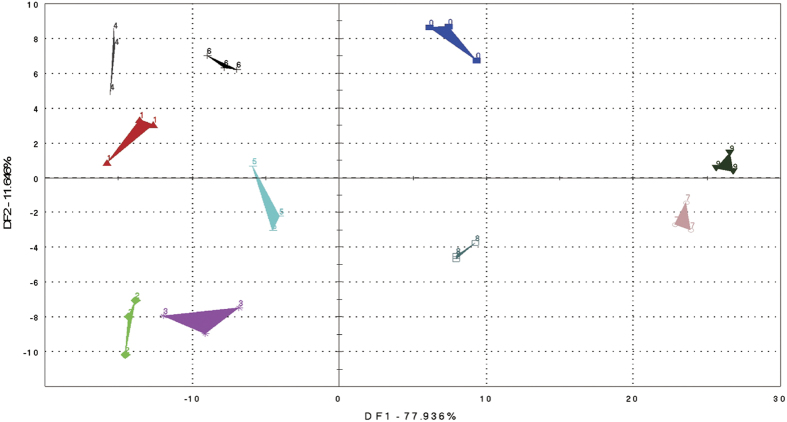
DFA map of 9 samples: six year aged liquor (0), control (1), 100 MPa-15 min (2), 100 MPa-30 min (3), 200 MPa-15 min (4), 200 MPa-30 min (5), 300 MPa-15 min (6), 300 MPa-30 min (7), 400 MPa-15 min (8), 400 MPa-30 min (9).

The DFA map of the 9 samples used in the study is shown in Fig. 2. As shown, the E-nose was able to distinguish the liquors samples based on the different treatment conditions. DF1 and DF2 explained 77.9% and 11.7% respectively, of the total variance. Thus, the first factor allowed effective discrimination of different liquor sample groups. As observed with PCA, samples 7, 8, 9 and 0 which referred to 300 MPa-30 min, 400 MPa-15 min, and 400 MPa-30 min treatment and six year aged liquor respectively, were positive on DF1, while other groups were negative. As a result, samples 7, 8, and 9 were clustered as one category, while samples 1 to 6 were clustered as a second category. There was also a difference between the six year aged liquor and samples 7, 8, 9 on DF2, also noted earlier with PCA.

The results of PCA and DFA analysis demonstrated that the high pressure treatments altered the volatile composition of fresh (young) liquors in comparison with the aged control. Furthermore, the isolation of samples 7, 8, 9 and 0 on both maps indicated that HP treatments at 300 MPa and 400 MPa played a more important role in influencing the parameters of aging of liquor than treatments at 100 MPa and 200 MPa. However, the results from PC2 and DF2 also demonstrated that some differences still existed between HP treated samples and the six-year aged liquor, indicating further exploration of the treatment effects &/or some level of aging would be needed following treatment. HP treated samples at 300 and 400 MPa were selected for further chemical analysis.

### Total acid content

**Figure 3:**
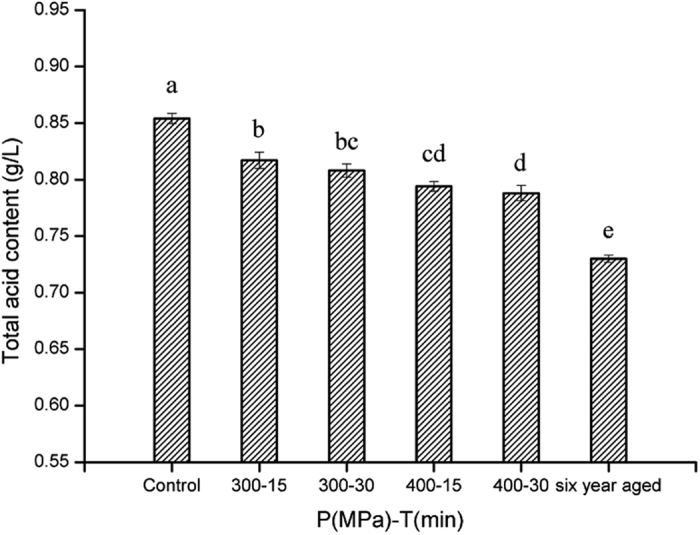
Total acid content of liquor samples treated with different conditions. The error bars indicate the standard deviation. Different letters above the bars indicate the significance under P < 0.05.

Total acid content of control, 300 MPa-15 min, 300 MPa-30 min, 400 MPa-15 min, 400 MPa-30 min and six-year aged samples, were 0.854, 0.817, 0.808, 0.794, 0.788 and 0.726 g/L, respectively, which were within the commercial specification range of Chinese liquors (0.6–1.58 g/L) (Fig. 3). Statistical analysis demonstrated that HP treatments contributed to lowering of the total acid content (p < 0.01). HP treatment at 400 MPa for 30 min yielded the lowest value of 7.7% total acid content, while a decline of 14.1% was observed in six year aged liquor. This reduction in acidity may have been caused by the loss of volatile acids and esterification reactions between acids and alcohol components during HP treatments and hence was considered as an indicator of the aging of liquor. Pereira *et al*.^20^ reported that most acids in TN sweet, TN dry and Malaysia wines decreased after aging for 1 month. Similar results were also reported by Pisarnitskii^21^. Overall differences between the four HP treated samples were not significant when they were considered together; however, differences existed between 300 MPa and 400 MPa HP treated samples (either for 15 or 30 min) indicating that pressure level could be a more effective factor than holding time in influencing the acid composition.

### Alcohol content

**Figure 4:**
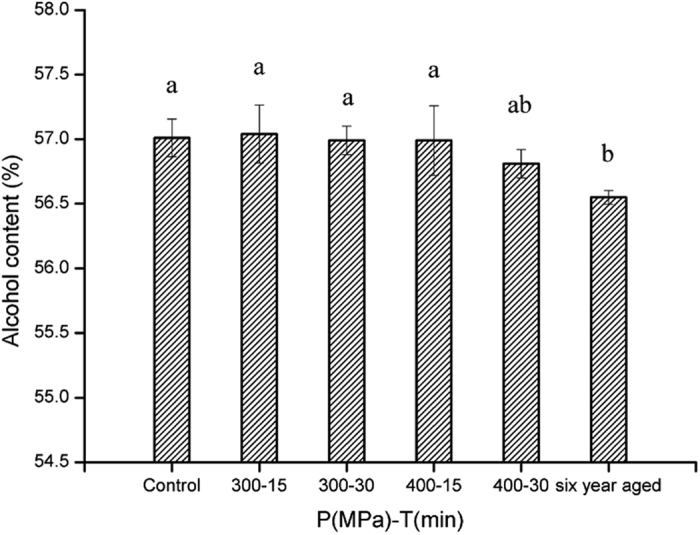
Alcohol content of liquor samples treated with different conditions. The error bars indicate the standard deviation. Different letters above the bars indicate the significance under P < 0.05.

A slight decline in alcohol content was observed in comparison with young liquor without HP treatment, with the lowest alcohol content obtained following HP treatment at 400 MPa-30 min (Fig. 4). The alcohol content of the aged sample was lower than all HP treated samples, although much closer to the 400 MPa-30 min treated sample. Some gaps still existed between the HP treated and aged samples with respect to the alcohol level. However, no significant decrease was found between untreated control and samples treated by HP (p > 0.05).

Traditionally, it is recognized that there will be some decline in the alcohol content during the natural aging process of liquors. Through the long time maturation, the odor peculiar to ethanol in spirits are reduced and, as a consequence, their tastes are altered to be favorable after aging. These have been linked to the changes in the structure of water and ethanol molecules^22^. Nose *et al*.^23^ linked the hydrogen-bonding structure of water-ethanol in aged whiskey to HNMR chemical shifts of the OH of water and ethanol. Furusawa *et al*.^24^ also reported that some volatile and nonvolatile compounds could assist the formation of ethanol-water clusters during the aging, which leads to the reduction of ethanol stimulation. Generally, in a wine sample, the aroma compounds represent only about 1 g/L, while the ethanol and water concentrations are about 100 and 900 g/L, respectively^25^.

### Total ester content

**Figure 5:**
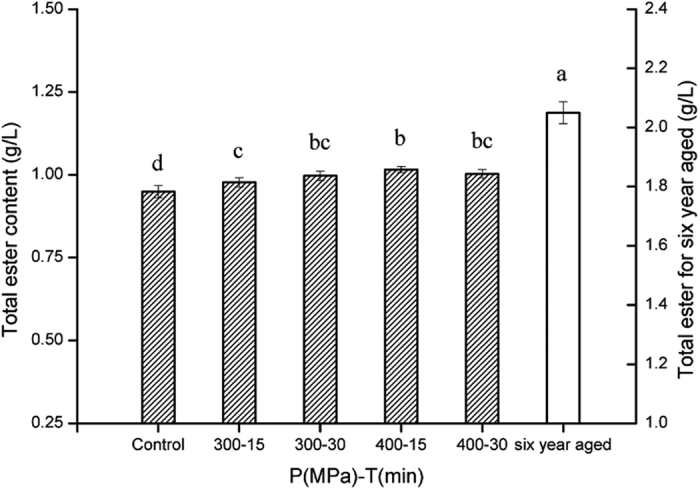
Total ester content of liquor samples treated with different conditions. The error bars indicate the standard deviation. Different letters above the bars indicate the significance under P < 0.05.

The total ester content of HP treated samples were significantly higher (Fig. 5), which also characterizes the liquor samples after the natural aging process. Total ester content of control, 300 MPa-15 min, 300 MPa-30 min, 400 MPa-15 min, and 400 MPa-30 min samples, were in the 0.95 to 1.02 g/L range. The highest level of total ester was obtained at 400 MPa-15 min with an increase of 5.7%. The total ester content of six year aged liquor was 2.053 g/L, which was twice the amount present in liquors without aging. Esters could be the most important class of all the aroma constitutes in Chinese liquor^2^. Trace component analysis indicates that more than 30 kinds of esters have been found in some famous brands of Chinese liquors, such as Maotai^3^,^26^, Daohuangxiang^27^ and Luzhoulaojiao^28^, and the total ester of the aged liquor could be as high as 2 g/L.

Esters are products of esterification reactions between acids and alcohols during the process of fermentation and aging, especially ethyl esters, which could be a reason for the decreasing in total acid and alcohol contents during aging. The enhancing effect of pressure on esterification reactions has been explained by the principle of Le Chatelier which states that any phenomenon accompanied by a decrease in reaction volume is enhanced by an increase in pressure, and vice versa^29^. As a result, HP treatment favors the increasing levels of total ester content in Chinese liquor.

### Total solid content

**Figure 6:**
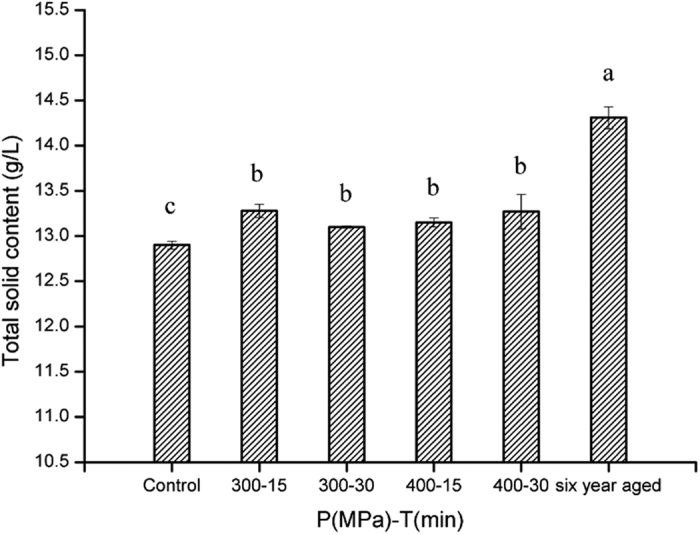
Total solid content of liquor samples treated with different conditions. The error bars indicate the standard deviation. Different letters above the bars indicate the significance under P < 0.05.

The total residual solid content represents the non-volatile substance &/or volatile substances of high boiling point. A significant increase (p < 0.05) was observed in the total solids content of HP treated samples (Fig. 6). Solid content is an undesirable index in commercial liquors, so a lower solid content is desired in high-quality liquors. The interaction between organic acid and metal ions during the manufacturing process is the main cause of the presence of solids. Qiao^30^ reported that metal ions, especially calcium and sodium ions, increased rapidly after aged for several years, which appeared to mainly dissolved from container. We hypothesize that the generation of esters of high boiling point following HP treatment might be responsible for the increase. However, further research is needed to get this substantiated.

### Gas chromatography analysis

In order to have a better understand on changes of total acid and total ester content, gas chromatography analysis was performed. As presented in Table 1, four acids and six esters were identified.

Acetic acid was found to be the most abundant aroma components of acid group with the concentration of 1131 mg/L in young liquor, while the total content of other three acids was below 100 mg/L. Generally, total acid content in Chinese liquor is usually expressed as the concentration of acetic acid, since more than 90% of the acidity is contributed by it. The concentration of acetic acid was 670 mg/L in six-year-aged liquor, with a decrease rate of 41% (p < 0.05) compared with young liquor. Meanwhile, a reduction of 15% (p < 0.05) was found in the concentration of acetic acid after high pressure treatment at selected condition (400 MPa-30 min). However, no significant change was observed in concentrations of propionic acid, butanoic acid and isopentanoic acid after high pressure treatment.

Ethyl acetate and ethyl lactate were the most prominent representatives in the ester group, with concentrations of 1656 and 1014 mg/L in young liquor, respectively. Concentrations of ethyl acetate in aged and high pressure treated liquors were 1904 and 1753 mg/L, respectively, which were significantly higher than in the young liquor. It appears that ethyl lactate is extremely stable during the aging and high pressure treatment process, since no significant change was observed in its concentration. High pressure treatment also caused an increase (p < 0.05) in the concentration of ethyl hexanoate from 11.5 mg/L to 55.7 mg/L. However, high pressure treatment had no effect on the concentration of ethyl butyrate (p > 0.05). Ethyl oenanthate and ethyl palmitate were, respectively, 54.6 and 82.2 mg/L, in the six-year-aged liquor, but those two components were not detected in young liquor and high pressure treated liquor.

### Sensory analysis

Sensory analysis is the most straightforward way to evaluate Chinese liquor quality because it would reflect the consumer perceptions. Therefore, liquors were subjected to quantitative descriptive analysis sensory tests to investigate the influence of HP treatment on sensorial characteristics of Chinese liquor. Liquors treated at 400 MPa-30 min were chose to conduct sensory analysis since this group performed well in electronic nose and chemical analysis. The testing procedure was based on the fact that some unpublished data reported the HP effects on flavor profiles of liquor to be transient and completely disappeared 2 days after the treatment.

**Figure 7:**
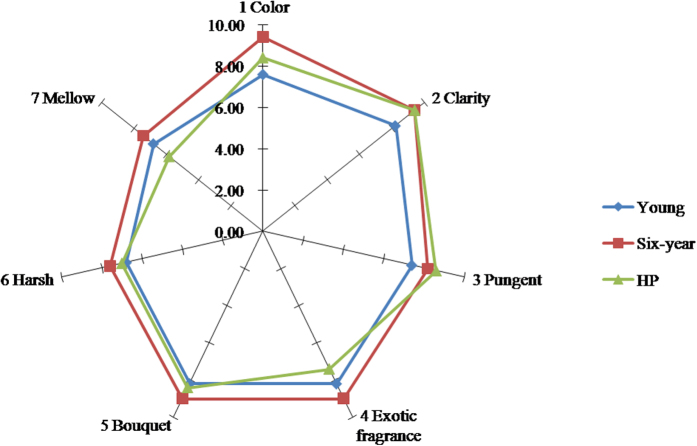
Effect of high pressure processing on the sensory characteristics of Chinese liquor.

The influence of HP treatment on Chinese liquor sensorial attributes is shown as a spider plot in Fig. 7. Attributes related to liquor appearance (Attribute 1 and 2) - the most dominant sales related factor - were found to be highly influence by HP treatment (p < 0.05), which demonstrated that changes of color and clarity induced by HP were clearly visible. As for the odor, a decline was found in the intensity of pungent smell (Attribute 3) after HP treatment (desirable). However, HP treatment also brought some exotic (external) fragrance (Attribute 4) to the liquor. This may have been caused by the packaging film used for subjecting the samples to the HP treatment (and possibly overcome using more appropriate packaging materials). Meanwhile, there was also no obvious change in Bouquet flavor (Attribute 5). The average score for HP treated liquor was significantly higher than the one for the fresh liquor (p < 0.05) for three parameters, except the taste (Table 2). Gustatory attributes were not statistically significant between fresh and HP treated liquors. However, a reduction in the intensity of mellow was observed after HP treatment. This could be the same reason as the formation of the external exotic smell, which could have been caused by the packaging bag. The overall quality of HP treated Chinese liquor was calculated by the average value of appearance, odor and taste (Table 2) which indicated the HP treated liquor scored higher than young liquor without treated (p < 0.05), while no significant difference was found between HP treated liquor and six-year aged liquor (p > 0.05) indicating the overall quality of the HP treated sample to be much closer to the aged liquor.

Overall, as can be expected, the six-year aged liquor scored highest for both color and flavor profiles (p < 0.05) of the liquor and HP treated samples had profiles much closer to the aged.

## Conclusions

The present study demonstrated that significant changes in aroma components resulted in Chinese liquor “Junchang” after HP treatment at selected conditions. E-Nose flavor profile analysis coupled with PCA and DFA confirmed that HP treatments at 300 MPa and 400 MPa were effective for causing desirable profile changes. Reducing trends were found both for total acid and alcohol content with HP treatment, and the lowest levels of total acid and alcohol content were obtained after 400 MPa-30 min treatment. HP resulted in a significant (p < 0.05) increase in total ester content of all test samples. These desirable findings were in accordance with natural aging process of liquor. A small undesirable increase was also observed in solid content, which might reduce the overall quality of liquor and might necessitate perhaps a filtration step. Results of GC analysis demonstrated that high pressure treatment caused a significant decrease in acetic acid, while ethyl acetate and ethyl hexanoate concentrations were increased after high pressure treatment. Sensory evaluation results showed that the overall quality of HP treated Chinese liquor was better than fresh ones, and average sensory scores placed the HP treated samples in between the fresh and aged, but more closed to the aged, thereby demonstrating a favorable transition to the aging process. Hence, the HP treatment has the potential to be a good adjunct for the aging Chinese liquor. Further studies may be desired before this technology can be implemented into commercial use.

## Materials and Methods

### Wine samples and HP treatments

Chinese liquor “*Junchang*”(sauce-flavor) was obtained from a local source and stored at room temperature before use. *Junchang* is a local brand produced by Junchang Liquor Factory, a winery in Sichuan province of China. This factory was founded more than ten years ago with an annual output of ~300 kiloliters in recent years. This brand is commercially not well recognized like the top 10 brands detailed earlier because it is a local brand and sold locally.

**Figure 8:**
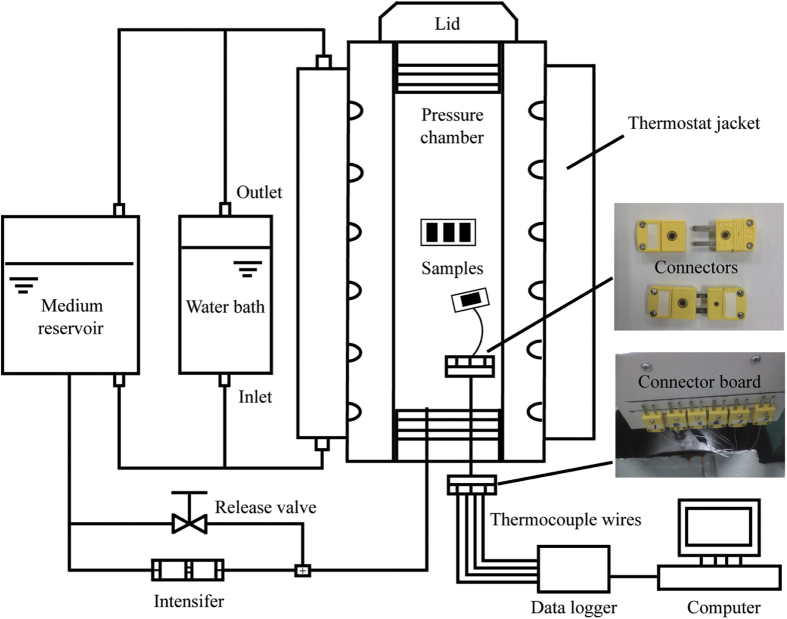
Schematic diagram of the high pressure experimental setup.

HP treatments were carried out using a laboratory scale high pressure equipment as shown in Fig. 8. The system consisted of a HP unit (UHPF-750, 5 L, Kefa, Baotou, China), was equipped with K-type thermocouples (Omega Engineering, Stamford, CT, USA) and a data logger (34970A, Agilent Technologies GMBH, Germany) for temperature measurement and a thermostat jacket connected to a water bath (SC-25, Safe, China) for maintaining the processing temperature. The intensifier used for generating the pressure was a batch type unit which built-up the pressure in a stepwise ladder-like process. Water was used as pressure transmitting medium (PTM) in this study, and the pressure vessel was maintained at 25 °C before pressurization. Sample temperature was monitored during the tests and was recorded at 1 s interval. Normally sample temperature is expected to increase by 3 °C every 100 MPa pressure rise due to adiabatic compression. However, to minimize this adiabatic heating, a low rate of pressurization was maintained (~100 MPa/min) so that the sample temperature easily equilibrated to the set point temperature of 25 °C. The pressure release time was kept less than 5 s. Different HP treatment combinations involving pressure level and time were used in the study: pressure (100, 200, 300 and 400 MPa) and holding time (15 and 30 min) and each combination was carried out in triplicate.

### Electronic nose analysis

A FOX model 4000 from AlphaMOS (Toulouse, France) was used as the E-Nose system. The FOX 4000 instrument consisted of eighteen metal-oxide-semiconductor (MOS) gas sensors, disposed in three controlled temperature chambers (Table 3). A generator of pure air (Whatman), attached to a dehydration cartridge filled with phosphoric anhydride was used for flushing the FOX. The multisensory array was interfaced with a computer which collected the sensor signals via an RS-232 port. A supervisor program (Alpha Soft Version 9) was equipped to control the whole system and analyse signals with chemometrics methods. The set-up was similar to the one used by Ragazzo-Sanchez *et al*.^31^.

A 10** **μL volume of liquor sample was diluted to 1000 μL using deionized double distilled water, and placed in a 10 mL headspace bottle and sealed by a bottle capper. The diluted liquor samples were kept at normal temperature for 1 h to let the aroma components volatilize sufficiently. A 2 mL volume of the headspace gas was then injected into the electronic nose for detection. Two pattern recognition methods, namely, the Principal Component Analysis (PCA) and the Discriminant Function Analysis (DFA) were used analyzing the signals gathered by electronic nose sensors.

### Chemical analysis

Total acid content, alcohol content, total ester content, and total solid content were measured according to standard Chinese liquor analysis methods^32^. All chemical reagents used were of analytical grade. The data of all groups were analyzed by one-way ANOVA at the 5% significance level using IBM SPSS statistics 21.0.

### Gas chromatography analysis

Gas chromatography analysis was performed using an Agilent 7890A gas chromatography (GC) system equipped with a flame ionization detector (FID). The column carrier gas was nitrogen at constant flow rate of 1 mL/min. Liquor samples were analyzed on a LZP-950 column (50 m × 0.32 mm i.d., 1.0 μm film thickness). A 1 μL sample was injected into the GC, and the split ratio was 1:1. The oven temperature was held at 65 °C for 8 min, then raised to 200 °C at a rate of 5 °C/min, and held at 200 °C for 50 min; injector and detector temperature were 230 °C and 250 °C, respectively.

The identification of aroma components in liquor samples was made by comparing the retention times with those of authentic compounds in a mixed standard solution. The quantification of aroma components was made based on calibration curves which were obtained with the mixed standard solution under the same chromatographic conditions as those used for liquor samples. All liquor samples were analyzed by direct injection method, replicated in five times and values were averaged. Only the liquor treated at 400 MPa-30 min was selected since this group performed well in electronic nose and chemical analysis.

### Sensory analysis

A panel of six judgers participated in the sensory evaluation. All panelists were trained to be familiar with the different quality attributes of typical Chinese liquors, according to the standard Chinese liquor analysis method^32^. A quantitative descriptive analysis method was used to determine the differences in the sensorial characteristics among HP treated, untreated and six-year aged Chinese liquors. All test samples were presented to every panelist after three days’ storage at room temperature following the HP treatment. Seven sensorial attributes associated with Chinese liquor mainly related to appearance, odor, taste and overall quality were selected by consensus. The definition of sensorial attributes and anchors used in quantitative analysis are shown in Table 4. For determining the overall sensory scores, the appearance, odor and taste parameters were individually averaged and then the combined average was taken in order to give equal weightage to appearance, odor and taste related quality parameters. The differences of the means were statistically tested as detailed earlier.

## Additional Information

**How to cite this article**: Zhu, S. M. *et al*. Effect of high pressure treatment on the aging characteristics of Chinese liquor as evaluated by electronic nose and chemical analysis. *Sci. Rep.* **6**, 30273; doi: 10.1038/srep30273 (2016).

## Figures and Tables

**Table 1:** Gas chromatography analysis.

**Components**	**Young**	**Six-year**	**HP**
Acetic acid	1131.5 ± 50.6a	670.2 ± 38.9c	969.6 ± 52.7b
Propanoic acid	15.8 ± 0.9a	14.9 ± 1.5a	13.1 ± 0.8a
Butanoic acid	41.4 ± 3.7b	55.7 ± 5.6a	45.5 ± 2.7b
Isopentanoic acid	28.8 ± 1.4a	25.9 ± 2.3a	25.6 ± 2.1a
Ethyl acetate	1656.6 ± 66.2c	1904.3 ± 50.7a	1753.8 ± 49.6b
Ethyl butyrate	8.2 ± 0.7b	17.5 ± 1.6a	7.4 ± 0.7b
Ethyl hexanoate	11.5 ± 1.2c	205.4 ± 10.2a	55.7 ± 3.3b
Ethyl lactate	1014.7 ± 50.3a	1053.2 ± 70.2a	1119.9 ± 66.9a
Ethyl oenanthate	ND	54.6 ± 3.2	ND
Ethyl palmitate	ND	82.2 ± 7.4	ND

Note: All values are means (mg/L) ± standard deviation (SD).

Values followed by different letters are significantly different (p < 0.05).

ND: not detected.

**Table 2:** Sensory analysis results.

**Attributes**	**Young**	**Six-year**	**HP**
Appearance	7.70 ± 0.67b	9.40 ± 0.51a	8.90 ± 0.56a
Odor	7.53 ± 0.63c	8.73 ± 0.59a	8.13 ± 0.63b
Taste	6.70 ± 0.48b	7.80 ± 0.42a	6.40 ± 0.69b
Overall quality	7.31 ± 0.59b	8.64 ± 0.51a	7.81 ± 0.63ab

Note: All values are means ± standard deviation (SD).

Values followed by different letters are significantly different (p < 0.05).

Overall quality was calculated by the average value of appearance, odor and taste.

**Table 3:** MOS Sensor name.

**Chamber**	**Number**	**Sensor**
Chamber CL	1	LY2/LG
	2	LY2/G
	3	LY2/AA
	4	LY2/GH
	5	LY2/gCTL
	6	LY2/gCT
Chamber A	7	T30/1
	8	P10/1
	9	P10/2
	10	P40/1
	11	T70/2
	12	PA2
Chamber B	13	P30/1
	14	P40/2
	15	P30/2
	16	P40/2
	17	T40/1
	18	TA2

**Table 4:** Definitions of sensorial attributes used in the descriptive analysis of Chinese liquor.

**Attributes**	**Definition**	**Anchoring points (full mark = 10)**
**Appearance**		
1. Color	Colorless and transparent	Colorless and transparent (10) – yellowish (0)
2. Clarity	Lack of cloudiness	Clear (10) – dull (0)
**Odor**		
3. Pungent	Strong and sharp; acrid	Imperceptible (10) – very intensive (0)
4. Exotic fragrance	Fragrance that doesn’t belong to liquor	Imperceptible (10) – very intensive (0)
5. Bouquet	Typical pleasing odor of liquor	Imperceptible (0) – very intensive (10)
**Taste**		
6. Harsh	Coarse; spicy	Imperceptible (10) – very intensive (0)
7. Mellow	Full and pleasing flavor	Imperceptible (0) – very intensive (10)
**Overall quality**	Overall quality of the sample	High quality (10) – inferior quality (0)
